# Cadmium alters whole animal ionome and promotes the re-distribution of iron in intestinal cells of *Caenorhabditis elegans*

**DOI:** 10.3389/fphys.2023.1258540

**Published:** 2023-09-26

**Authors:** Anuj Kumar Sharma, Lydia Finney, Stefan Vogt, Olena K. Vatamaniuk, Sungjin Kim

**Affiliations:** ^1^ Section of Plant Biology, School of Integrative Plant Science, Cornell University, Ithaca, NY, United States; ^2^ X-ray Science Division, Advanced Photon Source, Argonne National Laboratory, Lemont, IL, United States; ^3^ Department of Microbiology & Molecular Biology, Chungnam National University, Daejeon, Republic of Korea

**Keywords:** Heavy Metals, Cadmium, iron, Zinc transporters, copper transporters, ionome, XRF

## Abstract

The chronic exposure of humans to the toxic metal cadmium (Cd), either occupational or from food and air, causes various diseases, including neurodegenerative conditions, dysfunction of vital organs, and cancer. While the toxicology of Cd and its effect on the homeostasis of biologically relevant elements is increasingly recognized, the spatial distribution of Cd and other elements in Cd toxicity-caused diseases is still poorly understood. Here, we use *Caenorhabditis elegans* as a non-mammalian multicellular model system to determine the distribution of Cd at the tissue and cellular resolution and its effect on the internal levels and the distribution of biologically relevant elements. Using inductively coupled plasma-mass spectrophotometry (ICP-MS), we show that exposure of worms to Cd not only led to its internal accumulation but also significantly altered the *C. elegans* ionome. Specifically, Cd treatment was associated with increased levels of toxic elements such as arsenic (As) and rubidium (Rb) and a decreased accumulation of essential elements such as zinc (Zn), copper (Cu), manganese (Mn), calcium (Ca), cobalt (Co) and, depending on the Cd-concentration used in the assay, iron (Fe). We regarded these changes as an ionomic signature of Cd toxicity in *C. elegans*. We also show that supplementing nematode growth medium with Zn but not Cu, rescues Cd toxicity and that mutant worms lacking Zn transporters CDF-1 or SUR-7, or both are more sensitive to Cd toxicity. Finally, using synchrotron X-Ray fluorescence Microscopy (XRF), we showed that Cd significantly alters the spatial distribution of mineral elements. The effect of Cd on the distribution of Fe was particularly striking: while Fe was evenly distributed in intestinal cells of worms grown without Cd, in the presence of Cd, Fe, and Cd co-localized in punctum-like structures in the intestinal cells. Together, this study advances our understanding of the effect of Cd on the accumulation and distribution of biologically relevant elements. Considering that *C. elegans* possesses the principal tissues and cell types as humans, our data may have important implications for future therapeutic developments aiming to alleviate Cd-related pathologies in humans.

## 1 Introduction

Cadmium (Cd) is a highly toxic transition metal element that raises environmental concerns due to its toxicological effects and bioaccumulation features. Cd is increasingly emitted into our environment as industrial and consumer waste, stemming from the manufacturing of nickel-Cd batteries, pigments for paints, and the production of plastic, fertilizers, pesticides, insecticides, and more (Waalkes et al., 1992). Cd holds the seventh position out of 275 “Substance Priority List” by the Agency for Toxic Substances and Disease Registry (Registry, 2017), and has been classified as a human carcinogen by International Agency for Research on Cancer (IARC, 1993). Chronic exposure to Cd has been implicated in the induction of lung, prostate, kidney, and pancreatic cancer (Waalkes et al., 1992; Waalkes, 2000; Waalkes, 2003). Cd primarily enters the human body through food, water, and air followed by absorption and transport across the body (Murata et al., 1970). At the cellular level, Cd toxicity results from thiol capping of essential proteins, DNA damage (by interference with DNA repair processes), increased apoptotic events, and interference with the antioxidant defense system, leading to the generation of reactive oxygen species (ROS) (Stadtman, 1990; Waalkes et al., 1992; Waalkes, 2000; Waalkes, 2003; Valko et al., 2005; Clemens, 2006). Furthermore, Cd inactivates essential metalloenzymes by replacing endogenous metal co-factors from their binding sites (Waisberg et al., 2003). A recent study explained how Cd hijacks and mimics high Zn response by regulating a Zn-binding protein HIZR-1 (Earley et al., 2021). To deal with Cd toxicity, it is necessary to understand the exact mechanism of Cd uptake, transport, interaction with biomolecules, and its interaction with other metal ions in an organism as well as in cells, at precision.

Cd absorption can be mediated by transporters and channels for essential elements (e.g., iron [Fe], zinc [Zn], calcium [Ca], and manganese [Mn]) due to the similar ionic properties (Eide et al., 1996a; Clemens et al., 1998; Cohen et al., 1998; Levesque et al., 2008; Thevenod, 2010; Vesey, 2010; Sasaki et al., 2012). In rodents, the intestinal absorption of Cd is mediated by a divalent metal transporter-1 (DMT-1) that localizes to the apical membrane of enterocytes lining the small intestine (Bressler et al., 2004a). A low-specificity iron transporter from the ZIP family, IRT1 enables the entry of Cd from the soil into the plant roots (Eide et al., 1996a; Korshunova et al., 1999). After entering the cells, Cd can bind to proteins non-specifically, or to metallothionein (MT) or glutathione (GSH) (Kawata and Suzuki, 1983; Elinder et al., 1987; Zhou et al., 2006; Bozhkov et al., 2010; Hall et al., 2012; Wang et al., 2020).

When different dietary metals exist in a mixture, they can either act independently or interact with each other leading to synergistic or antagonistic effects. An antagonistic interaction between Zn and Cd with respect to their absorption and accumulation has been reported in plants (Tkalec et al., 2014). Cd treatment results in increased levels of Zn and Copper [Cu] in the rat glial cell line C6 leading to increased apoptosis, lipid peroxidation, and DNA damage (Nzen et al., 2012). Cd also disrupts Cu homeostasis in *Saccharomyces cerevisiae* by binding to the transcriptions factor Mac1, thereby reducing the expression of downstream target *CTR1*, which encodes a copper uptake transporter (Heo et al., 2010). Studies in plants have revealed that Cd alters the homeostasis of Fe and Cu, and mimics the transcriptional response caused by Fe or Cu deficiency (Besson-Bard et al., 2009; Wu et al., 2012; Gayomba et al., 2013). Overall, it is vastly accepted that Cd competes with the essential elements to enter the cell and Cd bioaccumulation could be accompanied by an imbalance of metal ions in living organisms (Eide et al., 1996a; Gunshin et al., 1997; Korshunova et al., 1999; Park et al., 2002; Vert et al., 2002; Hoch et al., 2012; Earley et al., 2021). The imbalance of metal ions in neuronal cells and the brain can cause neurodegenerative disorders like Alzheimer’s disease and Parkinson’s disease (Bush, 2000; Honda et al., 2005; Muhoberac and Vidal, 2013). Although, the molecular mechanism by which Cd enters the cell and how it is detoxified is substantially studied (Vatamaniuk et al., 2005a; Sooksa-Nguan et al., 2009; Schwartz et al., 2010a; Rakvacs et al., 2019) little is known about how it affects the broad range of essential and non-essential ions and their cellular distribution *in vivo*.

Here, using *C. elegans*, as a non-mammalian multicellular model system and a combination of inductively coupled–plasma-mass spectrometry (ICP-MS) analysis and synchrotron-based X-ray fluorescence microscopy (XRF), we discovered that exposure to Cd leads to a decrease in the internal concentration of biologically essential trace elements such as Zn, Mn, Cu, Fe, Co, while increasing the concentration of potentially toxic elements such as arsenic [As] and rubidium [Rb]. We also established that Zn homeostasis is important for basal Cd tolerance in *C. elegans*. Additionally, our studies revealed that Cd accumulates in punctate structures throughout the intestinal cells and significantly alters the distribution of Fe. Furthermore, we found that Fe and Cd co-localize in punctate structures. To the best of our knowledge, this is the first study to provide evidence of Cd’s impact on the ionome in a multicellular organism. Our findings can inform the development of therapeutics that correct, or delay the onset of diseases induced by Cd poisoning.

## 2 Materials and methods

### 2.1 *Caenorhabditis elegans* strains and growth culture condition

*C. elegans* strains were maintained at 20°C on Solid Nematode Growth medium (NGM) using the *Escherichia coli* OP50 strain as a food source as described (Kim et al., 2010). For ICP-MS analyses, NGM medium was supplemented with other trace elements (1X Mineral Mix) as detailed in supporting data (Supplementary Table S1) and was regarded as supplemental NGM (sNGM). *cdf-1(n2527), cdf-2(tm788), sur-7(ku119), ttm-1(ok3503)* single mutants and *cdf-1;cdf-2, cdf-1;sur-7, cdf-2;sur-7* double and *cdf-1;cdf-2;sur-7* triple mutants were the generous gift of Prof. Kerry Kornfeld (Washington University in St. Louis).

### 2.2 Heavy metal sensitivity assays

Heavy metal sensitivity assays were conducted following our established method (Schwartz et al., 2010a). Briefly, two adult hermaphrodites were placed on solid NGM plates supplemented with indicated concentrations of CdCl_2_, ZnCl_2,_ or CuCl_2_. Worms were allowed to lay eggs for 5 h to obtain several dozens of eggs and adult worms were removed to obtain age-synchronized animals. The hatched worms were grown for 3–4 days until the progeny of worms under control conditions reached the adult stage. Heavy metal sensitivity was assessed by comparing the percentage of progeny that have reached the adult stage under heavy metal vs. control conditions, and the morphological changes in intestinal cells were also compared using the Nomarski microscopy. Results are presented as mean values from three independent experiments each of which had three replicates. The total number of worms tested (n) is presented above each graph bar.

### 2.3 RNA isolation and RT-qPCR

The synchronized population of the ∼2,000 L1 stage worms was placed on 100 mm NGM agar plates seeded with *E. coli* OP50 bacteria and supplemented with or without 50 µM Cd. After 48 h, young adult hermaphrodites were collected from plates with M9 buffer and washed free from *E. coli* OP50 by two rounds of centrifugation (3,500 × g for 2 min). Worms, resuspended in M9 buffer were concentrated using Ultrafree-Cl Centrifugal Filter Units (MILLIPORE). Total RNA was isolated from worms with TRIZOL reagent (Invitrogen) according to the manufacturer’s recommendations. gDNA was cleared from the RNA preparations by DNAse (Roche) prior to the first strand cDNA synthesis. RT-qPCR was carried out as described earlier (Gayomba et al., 2013). Data were normalized to the expression of actin, *act-1.* The fold-difference (2^−ΔΔCq^) or relative quantities were calculated using the CFX Manager Software, version 1.5 (BioRad).

### 2.4 Functional complementation assays in *Saccharomyces cerevisiae*

*S. cerevisiae* strains used were DY1457 (α *ade6 can1 his3 leu2 trp1 ura3*), ZHY3 (DY1457 *zrt1*::*LEU2 zrt2*::*HIS3*) (Gitan et al., 2003). Cultures were grown in SD1 medium (0.67% yeast nitrogen base without amino acids) supplemented with auxotrophic requirements and indicated concentration of Cd.

### 2.5 Sample preparation for XRF

The synchronized population of the L1 stage worms were grown on NGM plates to adult stage. Synchronized young adult worms were collected and transferred to the 60 mm NGM plates seeded with OP50 and supplemented with or without 50 µM CdCl_2_. After 24 h, worms were collected and washed with excess S-basal (0.1M NaCl, 0.05 M KH_2_PO_4_, 5 mg/mL cholesterol) 3 times. Each time, worms were incubated in S-basal for 15 min before centrifugation to remove bacteria from the worm gut. Worms were finally filtered out with Ultrafree-Cl Centrifugal Filter Units (MILLIPORE) at 30 × g for 1 min to remove the remaining bacteria. Worms were then anesthetized with ice-cold 0.2% (w/v) NaN_3_ for 2 min. After all the worms were completely immobilized, they were washed twice with the de-icing agent, ice-cold 1.5% (w/v) CH_3_COONH_4_. Immobilized animals were transferred to 200 nm thick silicon nitrite window (SiMPore, Inc.) and straightened using an eyelash. Excess liquid was removed using fine-tapered paper wick (MiTeGen, United States). Then the window with attached worms was plunge-frozen in liquid nitrogen slush and dried overnight using a Balzers High Pressure Freezer. Freeze-dried worms were stored in a cryovial at room temperature with desiccants.

### 2.6 XRF

Dehydrated samples were analyzed by XRF using the X-ray microprobe at 2-ID-E beamline in the Advanced Photon Source (Argonne, IL). An incident energy of 10.5 KeV with a dwell time of 0.1 s for stepsize of 0.5 µm, to excite the Cd L-edge. Full X-ray emission spectra was collected by Vortex 4-element silicon drift detector (SII, Inc.) to obtain the information of S, Cl, K, Ca, Mn, Cu, Fe, Zn, Co as well as Cd. Elemental maps were obtained and quantified using MAPS software (GLOWACKI, 2020).

### 2.7 Worm preparation and ICP-MS analysis

Worms were grown on OP50 seeded sNGM plates containing ×1 mineral mix (Nass and Hamza, 2007) (to make sure a detectable amount of each element). The sNGM was also supplemented with the indicated concentrations of CdCl_2_. Worms were harvested on fourth day of growth and washed free from *E. coli* OP50 by several rounds of washing and centrifugation as described above. The worm culture was thoroughly mixed and transferred into plastic reservoirs so that they could be pipetted using multichannel pipettes. Meanwhile, a 350 µL AcroPrep™ 96 filter plate with prefilter material/1.2 µm Supor^®^ membrane (Pall Life Sciences) was wetted with methanol (300 µL/well) and washed with DI water (400 µL/well). The *C. elegans* cultures were then pipetted into the filter membrane plate (100 µL/well, four replicates per mutant line, i.e., ∼1,000 worms per well), and washed and rinsed with EDTA solution (1 mM, pH 8.0) and DI water, respectively. A total of four such wash and rinse steps were performed (350 µL/well). Note that the filtration step separates the worms from the bacteria feed as the bacteria go through the membrane pores as well. Further, the filter membrane plate was dried (150 min) in an oven at 88°C. Nitric acid (45 µL/well) was added to the dried worms in the filter plate and the samples were digested in a heating block set at 88°C for about 60 min. The digested samples were drawn into a deep-well collection plate containing 0.025% Triton X-100 solution (95 µL/well using vacuum manifold). Deionized water (135 µL/well, 3 times) was then drawn through the filter membrane and also into the collection plate; thus the final solution volume per well in the plate was 500 µL (with final Triton X-100 concentration of 0.005%). Sample solutions were thoroughly mixed and analyzed using a Perkin Elmer DRC II ICP-MS with the ESI (Elemental Scientific, Inc.) SC-2 auto-sampler and the Apex Q sample introduction system. Calibration standards were prepared from single elemental stock solutions containing all the elements of interest (Na, Mg, Al, P, S, Cl, K, Ca, Mn, Fe, Co, Ni, Cu, Zn, As, Se, Mo, Cd, Rb, and Sr). The standards were matrix-matched (i.e., contain Triton X-100 and nitric acid). Note that the Triton X-100 was added to enable smooth self-aspiration of the PFA nebulizer of the Apex Q. The instrument software uses linear calibration to determine the concentrations of the individual elements in the digested *C. elegans* samples. These concentrations were used together with the dilution factor and the calculated sample weights to determine the elemental concentrations (ppm or molarity) in the original *C. elegans* samples.

### 2.8 Statistical analysis

Statistical analysis of data was performed using One-way ANOVA and Student’s t-test depending on the experimental requirements. Sample size (n) is indicated in the Figure legends. Error bars indicate ± S.E.M. Statistical significant differences are indicated with asterisks (*, *p* ≤ 0.05; **p ≤ 0.01).

## 3 Results

### 3.1 Whole animal (*Caenorhabditis elegans*) ionome and Cd-mediated alteration in the ionome

Due to the important role of essential and non-essential elements in growth and development, ionomics has become a common practice for establishing the nutritional value of food and the health of animals (Parent et al., 2013; Ma et al., 2015; Zheng et al., 2016). The ionome of plant leaf and unicellular yeast has been found changed according to physiological state, genetic background, and media type (Eide et al., 2005; Baxter et al., 2008). Recently, the ionome of different organs from 26 mammalian species has been analyzed showing a correlation with body mass and longevity (Ma et al., 2015). However, little is known about how the ionome of a whole animal responds to changes in its surrounding environment. To study the effect of Cd exposure on the *C. elegans* ionome, we first determined the normal concentration of 20 elements, including essential macro- and micro-nutrients, and non-essential potentially toxic elements in Bristol wild-type (N2) worms using ICP-MS (Table 1). To allow the detection of trace metals (e.g., [Cu], [Mn], nickel [Ni], [Co], selenium [Se]) as well as potentially toxic elements (e.g., [As], [Cd], [Rb]), we used supplemented-nematode growth medium (sNGM), by adding each element at concentrations as described in Supplementary Table S1, adapted from Nass and Hamza (2007). Additionally, we also spiked *E. coli* OP50 culture (used to seed sNGM plates) with the same mineral mix as described above to keep the elemental concentration uniform in treatment. This concentration did not affect the growth of wild-type worms and sNGM was only used for worm cultivation during ICP-MS assays. Analysis using ICP-MS revealed that the accumulation of various elements varied, spanning several orders of magnitude, depending on the element and its function as a macro- or micro-nutrient. For example, worms accumulated 10,883.5 ± 787.9 ppm of the macronutrient phosphorus [P] but 72-fold less of the micronutrient Cu (149.7 ± 13.0 ppm) and 9,302-fold less of another micronutrient Co (1.17 ± 0.2 ppm) as compared to P (Table 1). Cd accumulation was the lowest (0.27 ± 0.01 ppm) among all elements. On the other hand, the concentration of another non-essential and toxic element, As was higher than that of several established micronutrients or beneficial elements such as Ni, Co, and Mo (Table 1). The overall accumulation pattern, from highest to lowest, was as follows (from highest to lowest accumulation): P > K > S > Ca > Mg > Cl > Na > Zn > Mn > Fe > Cu > Se > Al > As > Sr > Ni > Co > Rb > Mo > Cd (Table 1). Comparison of concentrations of these elements in *C. elegans* vs. their levels in the growth medium (before growing worms) revealed that Cl, Na, and Mo were accumulated to only 30%, 35%, and 46% of the media levels, respectively (Table 1). Notably, the concentration of toxic elements As and Cd were 19 and 54 fold higher than in the growth medium. The baseline concentration of As and Cd in basal media was 1 ppm and 0.05 ppm, respectively. This strongly suggests the existence of mechanisms for their uptake, accumulation and intracellular retention in worms.

**TABLE 1:** The concentration of elements in wild-type (N2) *C. elegans* and sNGM media.

Element	Conc., in ppm (worm body)	Conc., in ppm (media)
Al27	36.9 ± 4.7	0.500
As75	19.4 ± 0.4	1
Ca44	2124.8 ± 259.5	75.2
Cd111	0.27 ± 0.01	0.05
Cl35	623.8 ± 47.6	2104.7
Co59	1.17 ± 0.2	0.05
Cu65	149.7 ± 13.0	4
Fe57	209.4 ± 13.4	2.9
K39	7147.7 ± 262.2	1069.6
Mg25	1204.9 ± 88.0	90.6
Mn55	347.7 ± 39.4	8.3
Mo95	0.46 ± 0.02	1
Na23	467.7 ± 26.9	1351.2
Ni60	1.26 ± 0.17	0.4
P31	10883.5 ± 787.9	775
Rb85	1.02 ± 0.02	0.4
S34	3818.0 ± 200.1	23.55
Se82	48.7 ± 5.6	5
Sr88	14.2 ± 1.7	0.4
Zn66	350.2 ± 34.4	7.26

The concentrations of elements in media in the right column are nominal, and not measured by ICP-MS. Data represent mean values + S.E. from four independent experiments.

**FIGURE 1:**
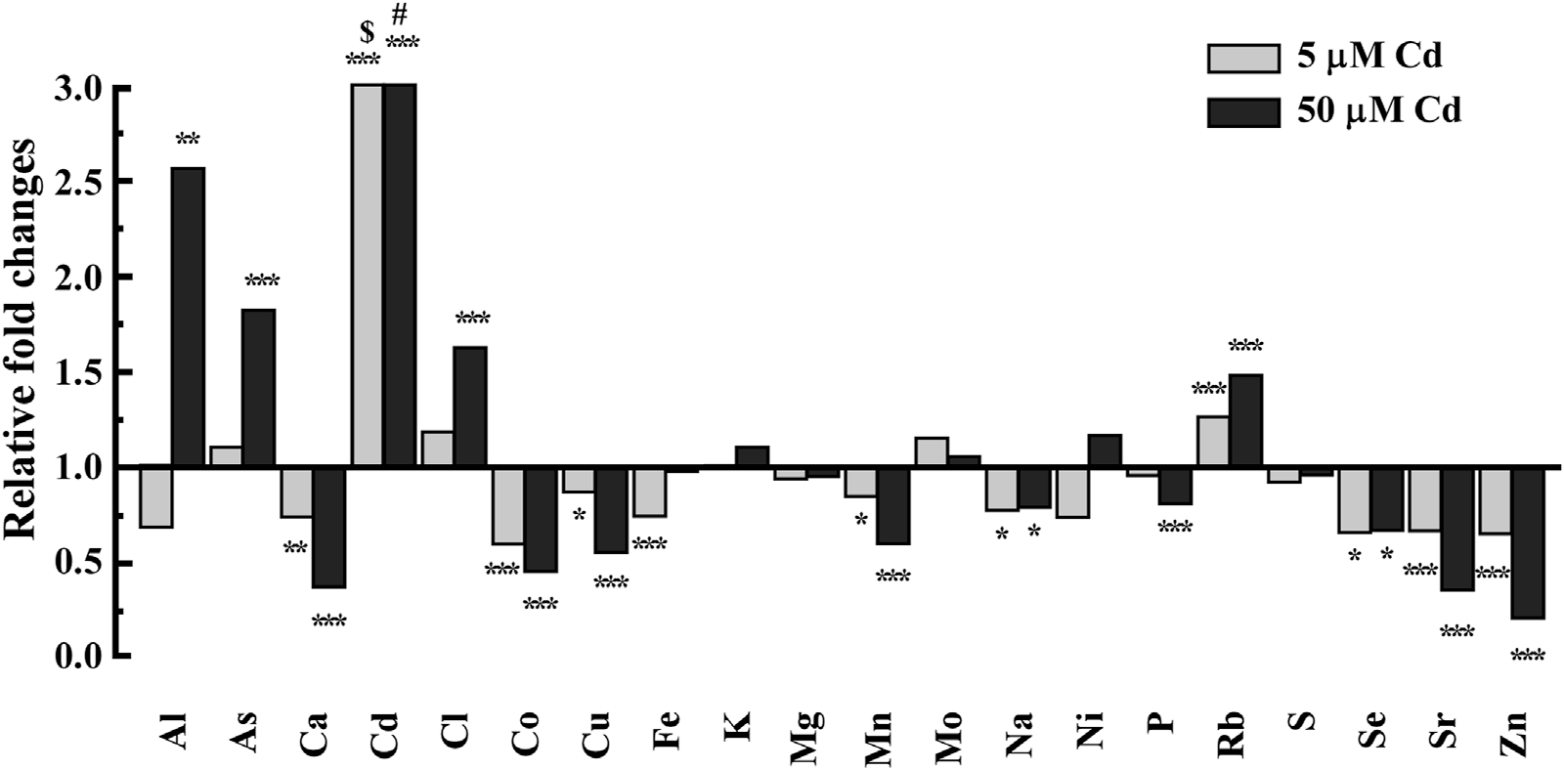
ICP-MS-based analysis of the effect of Cd on accumulation of essential elements in the wild-type worms. Worms were grown on NGM-plates supplemented with mineral elements (refer to as sNGM) in the absence or presence of Cd at the indicated concentrations. CdCl_2_-treated conditions were normalized to control conditions (no CdCl_2_ treatment). Control conditions are designated as 1 in the y-axis. $ and # denotes 253, 1,497 fold changes of Cd, respectively. Error bars indicate ±S.E.M. (*n* = 4). Note that error bars are very small and are not visible.

To determine the effect of Cd on the *C. elegans* ionome, we compared the concentrations of different elements in wild-type worms grown on control sNGM media and those grown on sNGM medium supplemented with 5 or 50 μM Cd. 5 μM Cd was chosen because earlier studies showed that this concentration does not alter the growth and development of the wild-type worms but this is the highest concentration that Cd-sensitive *hmt-1* mutants can tolerate (Vatamaniuk et al., 2005a; Schwartz et al., 2010a) whereas 50 μM Cd is the highest concentration tolerated by the wild-type worms with minimum signs of toxicity (Vatamaniuk et al., 2005a; Schwartz et al., 2010a). We noted that Cd achieved the biggest fold accumulation difference (253.85 fold & 1,497.12 fold respectively) in worms grown on Cd-containing media compared to worms grown on basal medium resulting in significant changes of the *C. elegans* ionome (Figure 1). Especially, the concentrations of essential elements such as Zn, Mn, Cu, Ca, and Co were significantly decreased, while the concentrations of potentially toxic elements such as As and Rb were significantly increased compared to control (Figure 1). This pattern was established by normalizing Cd-treatment values with control values for each element. We regarded these changes in the internal ion accumulation pattern as the ionomic signature of Cd toxicity in *C. elegans*. We speculate that Cd toxicity in worms arises not only from increased Cd accumulation but also from Cd-induced alterations in the ionome.

### 3.2 Zn homeostasis is essential for basal Cd resistance in *Caenorhabditis elegans*

**FIGURE 2:**
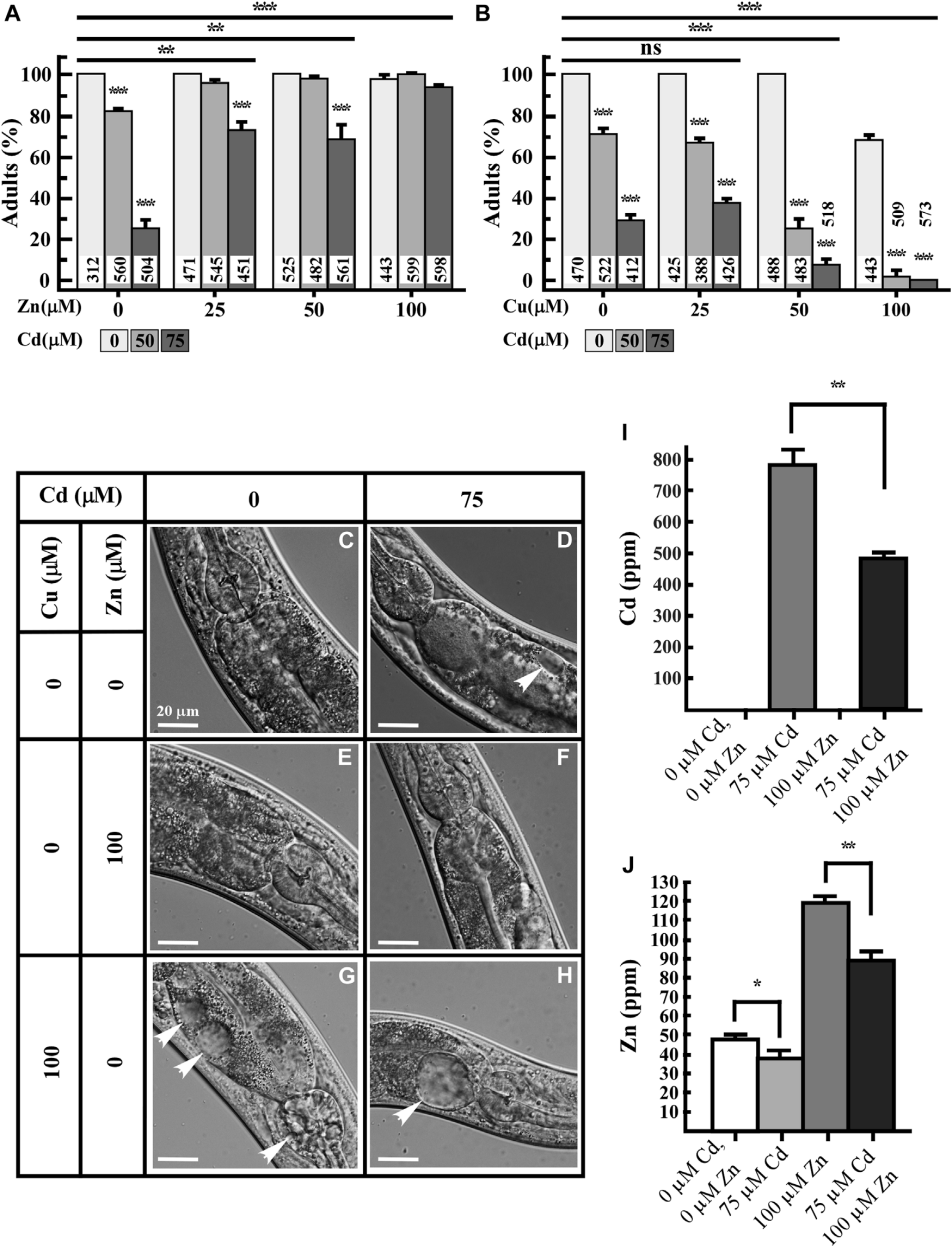
Zn rescues while Cu increases Cd toxicity of worms. **(A,B)** Percentage of worms that reached adult stage after 3.5 days of growth in the absence or presence of the indicated concentrations of Cd, Zn, Cu or both CdCl_2_ and ZnCl_2_ or both CdCl_2_ and CuCl_2_. Number of worms tested are indicated. **(C–H)** Differential interference contract (DIC) micrographs of intestinal cells of 5-day-old worms grown under indicated concentrations of CdCl_2_, ZnCl_2_ or CuCl_2_. Arrow heads indicate necrosis induced by indicated heavy metal treatment. **(I,J)** ICP-MS analysis of the concentration of Cd **(I)** and Zn **(J)** in worms, grown on solid NGM supplemented with the indicated concentrations of Cd or Zn, or both metals simultaneously (*n* = 3 independent experiment). Error bars indicate ± S.E.M. Statistically significant differences are indicated with asterisks (*p < 0.05; **p < 0.01).

Our ICP-MS-based studies suggest that, in addition to hyper-accumulation of Cd, the decreased accumulation of essential elements and increased accumulation of toxic elements in Cd-grown worms might contribute to Cd toxicity. If this is the case, supplementing the NGM with essential elements, e.g., Zn and/or Cu could potentially reduce Cd toxicity. Zn and Cu are critical micronutrients and known to exhibit synergistic or antagonistic effects with Cd (Pence et al., 2000; Liu et al., 2008; Nzen et al., 2012; Tkalec et al., 2014; Earley et al., 2021). To test our predictions, we supplemented the medium containing Cd with either Zn or Cu and assessed Cd toxicity in worms. Cd toxicity was evaluated based on the ability of the animals to reach the adult stage and the presence of internal morphological changes, as described previously (Vatamaniuk et al., 2005a). We observed that while 100% of animals reached the adult stage when grown on the NGM without Cd, their development was significantly delayed in the presence of Cd. Specifically, only 82% ± 1.5% and 30% ± 5.4% of animals reached adulthood with 50 or 75 µM Cd treatment, respectively (Figure 2A). Also, worms treated with 75 µM Cd for 4.5 days developed necrotic lesions in intestinal cells (Figure 2D). In contrast to Cd, the supplementation of Zn did not affect the growth or cellular morphology of worms (Figures 2A, E). Control worms grown on regular NGM media show healthy intestinal cells (Figure 2C). Importantly, the addition of Zn rescued the growth and morphological defects observed in Cd-treated worms (Figures 2A, F). The ability of Zn to rescue Cd sensitivity in worms can be explained by two hypothesis: 1) Zn competes with Cd for the uptake leading to a decreased internal concentration of Cd, and 2) Zn and Cd are taken up independently, and additional Zn is needed for protecting Zn-requiring coenzymes (e.g., enzymes that use Zn as a cofactor) from Cd toxicity. To test these hypotheses, we analyzed the concentrations of Zn and Cd in worms that were grown with Cd, Zn or both added to the sNGM media. Consistent with our previous findings (Figure 1), Cd accumulation in worms was associated with a decrease in Zn concentration (Figure 2J). Also, Zn supplementation decreased Cd concentration in worms by 0.62-fold compared to those grown in the presence of Cd alone (Figure 2I), suggesting overlapping uptake pathways for Zn and Cd. Similarly, the Zn concentration in worms treated with Zn and Cd simultaneously was 0.74-fold lower than in worms treated with Zn alone (Figure 2J), however, the Zn concentration was 1.85-fold higher in worms grown with both Zn and Cd compared to control conditions (Figure 2J). Notably, Cd concentration was still relatively high, reaching up to 490 ppm in worms treated with both Cd and Zn, compared to near-zero levels in non-treated control worms. Nevertheless, the majority of worms reached the adult stage, implying that the additional Zn plays a role in Cd detoxification (Figure 2I).

**FIGURE 3:**
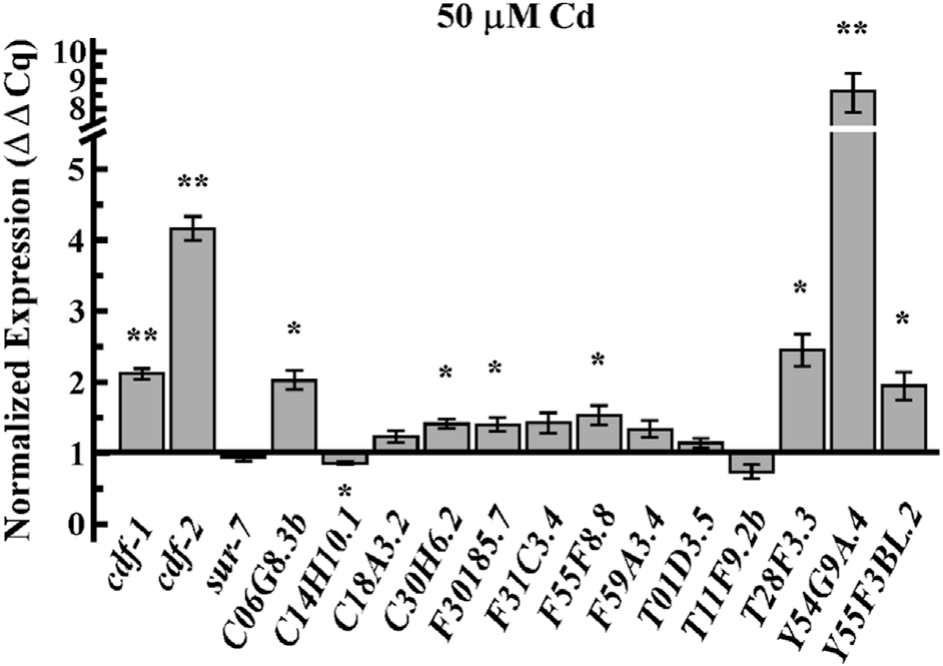
Cd upregulates mRNA expression of Zn transporters. qRT-PCR analysis of transcript abundance of members of the CDF family, *cdf-1, cdf-2*, and *sur-7* and the Zip family, *C06G8.3b, C14H10.1(zipt-13), C18A3.2(zipt-3), C30H6.2(zipt-17), F30185.7, F31C3.4(zipt-1), F55F8.8, F59A3.4(zipt-11), T01D3.5 (zipt-9), T11F9.2b, T28F3.3(zipt-7.1), Y54G9A.4 (zipt-2.3), Y55F3BL.2(zipt-15)* in adult worms treated with 50 µM of CdCl_2_. Results are normalized to the expression of *act-1* and are presented relative to the expression of genes in worms grown under control condition, which is designated as 1. Error bars indicate ±S.E.M. (*n* = 6). The asterisks represent statistically significant differences between mean values of positive control, at 0 µM CdCl_2_ and Cd treated conditions (*p ≤ 0.05 and **p < 0.001, respectively).

To test whether the effect of Cd on Zn homeostasis is associated with the distribution of Zn uptake and/or internalization, we examined the expression of Zn transporters in Cd-treated worms. Members of two major families of transporters are involved in Zn homeostasis in animals: the Cation Diffusion Facilitator (CDF/ZnT/SLC30) family and the Zrt, Irt-like proteins (Zips/SLC39) family (Eng et al., 1998; Guerinot, 2000; Eide, 2004). The *C. elegans* genome encodes 14 ZIP and 14 CDF family members. Next, we tested the effect of Cd on the expression of some of these genes. Quantitative real-time PCR (qRT-PCR) analysis revealed that the expression of *cdf-1, cdf-2* among CDF family, and most of Zips family [with the exception of *C14H10.1 (zipt-13)* and *T11F9.2b*], were significantly upregulated by Cd treatment (Figure 3). This experiment suggests the role of Zn-transporters in encountering Cd-toxicity.

Together, these results indicate that Zn homeostasis is important for basal Cd resistance in *C. elegans* through the ability of Zn to decrease internal Cd accumulation by competing for uptake, and presumably by protecting internal Zn-requiring cellular proteins against Cd toxicity.

### 3.3 Cu increases Cd-caused toxicity in *Caenorhabditis elegans*

Cd treatment also affects Cu homeostasis in *Saccharomyces cerevisiae*, *Arabidopsis thaliana,* and *Drosophila melanogaster* regulating the expression of genes encoding copper transporters, *CTR1*, *COPT2*, and Ctr1B, respectively (Balamurugan et al., 2009; Heo et al., 2010; Gayomba et al., 2013). Because the exposure to Cd also decreased Cu accumulation and the Cu homeostasis was shown to be important in maintaining basal tolerance to Cd in *Arabidopsis thaliana* (Gayomba et al., 2013), we examined if supplementation with Cu would also rescue Cd-caused toxicity in worms. Therefore, we grew worms with increasing concentrations of either Cu or Cd or both. We found that while supplementing media with lower concentrations of Cu (25 and 50 µM) did not affect the worm growth and cellular morphology, treatment with 100 µM Cu delayed the development (∼70%) and caused necrosis of intestinal cells even without Cd (Figures 2B, G). This confirms that Cu is also toxic to worms at higher concentrations (Yuan et al., 2018; Chun et al., 2017). Furthermore, when Cu and Cd were added simultaneously, Cu exacerbated Cd toxicity, causing a significant decrease in number of worms reaching adulthood and severe necrosis of intestinal cells (Figures 2B, H). Approximately 70% of worms reached the adult stage in the presence of 50 µM Cd + 25 µM Cu whereas only about 25% worms reached the adult stage in the presence of 50 µM Cd + 50 µM Cu, though 100% of worms treated with 50 µM Cu reached the adult stage in the absence of Cd (Figure 2B). Treatment of 100 µM Cu + 50 or 75 µM Cd delayed the growth significantly not allowing more than 1% worms to reach the adult stage (Figure 2B). There are 15 candidate proteins transcribed from 10 gene loci that showed higher orthology to human hCtr1 (Kim et al., 2008), among those, *CHCA-1 (F58G6.9)/hCTR1* is responsible for Cu uptake into worm intestine (Yuan et al., 2018). *CUA-1* is the only ortholog to human Cu exporter ATP7A/B and also functions in Cu homeostasis in worms (Chun et al., 2017). RT-qPCR analysis revealed that *chca-1* and its closest homologs (*F58G6.7*, *F58G6.8*) were significantly downregulated by Cd along with *K12C11.3* but two of the candidates were upregulated, i.e., *F27C1.2* and *F31E8.4* (Supplementary Figure S1), nevertheless, *CUA-1* expression was unaltered (Supplementary Figure S1). Past studies have shown that *CHCA*-1 downregulation leads to a decreased Cu accumulation in worms and that *CUA*-1 does not respond to Cu concentration at the transcriptional level (Yuan et al., 2018; Chun et al., 2017). By contrast, studies in plants, have shown that Cd toxicity increases the expression of hCTR1 homologs, *COPT1*, *COPT2*, and *COPT6,* and stimulates Cu uptake (Gayomba et al., 2013). So, our findings are consistent with existing studies on the crosstalk between Cu homeostasis and Cd resistance. However, while in plants, Cu is thought to protect from Cd toxicity, Cu might exacerbate Cd toxicity in animals. We speculate that the decreased Cu accumulation in Cd-cultured worms (Figure 1) may be serving a protective function to reduce the cellular damage caused by Cd toxicity.

### 3.4 Zn transporters are involved in Cd resistance in *Caenorhabditis elegans*

Earlier studies showed that in addition to *cdf-1*, *cdf-2* and *sur-7, ttm-1,* another member of the CDF family was also transcriptionally highly activated by Cd (Huffman et al., 2004; Nass and Hamza, 2007). We next tested whether the function of CDF-1, CDF-2, SUR-7 and TTM-1 is required for basal Cd resistance in *C. elegans*. To do so, we examined Cd sensitivity of worms that lack one or more of these transporters. *cdf-1, sur-7* and *ttm-1* mutants were as sensitive to Cd as either of the single mutants, except for *cdf-2*, implying that these genes share a common pathway to confer Cd resistance. To further test the function of Zn transporters in Cd resistance, we took advantage of a heterologous yeast system. In *S. cerevisiae*, Zn uptake is primarily mediated by *Zrt1* and *Zrt2* transporters (Gitan et al., 2003). We grew WT (wild type) and ZHY3 (*zrt1; zrt2*) yeast cells on different concentrations of Cd and found that ZHY3 is more sensitive to increased concentrations of Cd than WT (Supplementary Figure S2). This also suggests that in the absence of Zn importers (*zrt1;zrt2*), cells cannot uptake enough cellular zinc to counter Cd toxicity. Taken together, we conclude that Zn homeostasis is essential for basal Cd resistance.

### 3.5 Cd alters the concentration and distribution of trace elements

The ICP-MS-based studies enabled us to quantify concentrations of different elements including Cd, and the effect of Cd on the equilibrium of physiologically relevant, as well as potentially toxic metals (Figure 1). While these analyses are very informative, they do not provide information about the spatial distribution of Cd and other elements at cellular levels. It is possible that the spatial distribution of Cd and its effect on the distribution of other elements might be among the underappreciated bases for Cd-caused diseases.

To analyze the spatial distribution of Cd and other elements in worms we employed X-ray fluorescence microscopy (XRF), which has become the method of choice for visualizing the distribution of transition metals *in situ* (Finney et al., 2007; Tian et al., 2011). For comparative analysis, young adult worms were grown on 0 or 50 μM Cd containing NGM plates for 24 h and then subjected to XRF analyses. One of the primary tissues in *C. elegans*, the intestine plays a crucial role in the digestion and assimilation of food as well as the production and storage of miro- and macromolecules. *C. elegans*’ intestines are comprised up of 20 large epithelial cells that are arranged in bilaterally symmetric pairs to form a long tube that encloses a lumen (Figure 5A, McGhee, 2007). We found that the bulk of Cd was localized in intestinal cells in animals treated with Cd, where it exhibited a punctate distribution with peaks in the vicinity of nucleus-like structures (Figures 5B, D). Consistent with the results from the ICP-MS study (Figure 1), Cd exposure correlated with a decreased concentration of biologically essential elements such as Ca, Cu, Zn, Mn, Co (Figure 5B, compare upper and lower panels). In addition, the concentration of S, K, Cl was also reduced in Cd-grown vs. control worms (Figure 5B, compare upper and lower panels). While Cd did not alter the spatial distribution of the majority of analyzed elements, profound differences were found in the spatial distribution of Fe (Figure 5C). Whereas Fe was localized evenly at low concentrations through the entire intestine of worms grown under control conditions, it accumulated at a high concentration in discrete structures resembling the nucleus of intestinal cells (Figure 5C). Furthermore, a large portion of Fe and Cd co-accumulated as indicated by arrows in intestinal cells (Figure 5D).

Taken together, these results suggest that Cd affects the *C. elegans* ionome by lowering the concentration of diverse trace elements and contributes to the altered spatial distribution of Fe in intestinal cells.

## 4 Discussion

Cd has been at the center of heavy metal toxicity research since Pb-containing petrol was banned and Hg/As containing products were restricted in developed countries because of their high toxicities (Tchounwou et al., 2012). Due to its wide commercial usage, such as in batteries and its bio-accumulative nature, Cd continues to pose a threat to human health (Registry, 2017). Consequently, efforts have been made to understand the mechanism of Cd uptake, toxicity, and detoxification (Meshitsuka et al., 1987; Vatamaniuk et al., 2005a; Occupational Medicine Forum, 2006; Cui et al., 2007; Sooksa-Nguan et al., 2009; Schwartz et al., 2010b; Moulis, 2010; Matovic et al., 2011; Tvermoes et al., 2011; Aquino et al., 2012; Hall et al., 2012; Hartwig, 2013; Luckett et al., 2013; Marchetti, 2013; Person et al., 2013). Particularly, the roles of Zn, Ca, Fe, and Mg have been extensively studied in Cd uptake as transporters of these elements are commonly exploited by Cd in biological systems (Hinkle and Osborne, 1994; Bouckaert et al., 2000; Cui et al., 2004; Bergeron and Jumarie, 2006; Ohana et al., 2006; Levesque et al., 2008; Thevenod, 2010). In this study, we established the ionomic signature of Cd toxicity in *C. elegans*. This is the first evidence showing the effect of Cd on the ionome in a multicellular organism. We demonstrate that the inherent sensitivity of worms to Cd is not only due to the increased Cd accumulation but also due to the decreased amounts of essential elements such as Zn, Fe, Mn, Co, Cu, and increased accumulation of toxic elements such as As and Rb. Fe, Zn, and Cu are essential elements, required by different enzymes and transcriptional factors as co-factors. Deficiencies or excesses of Fe, Zn, and Cu have been associated with multiple disease conditions in humans (Kim et al., 2008; Hansch and Mendel, 2009; Camaschella and Strati, 2010; Chasapis et al., 2012; Mocc et al., 2012).

**FIGURE 4:**
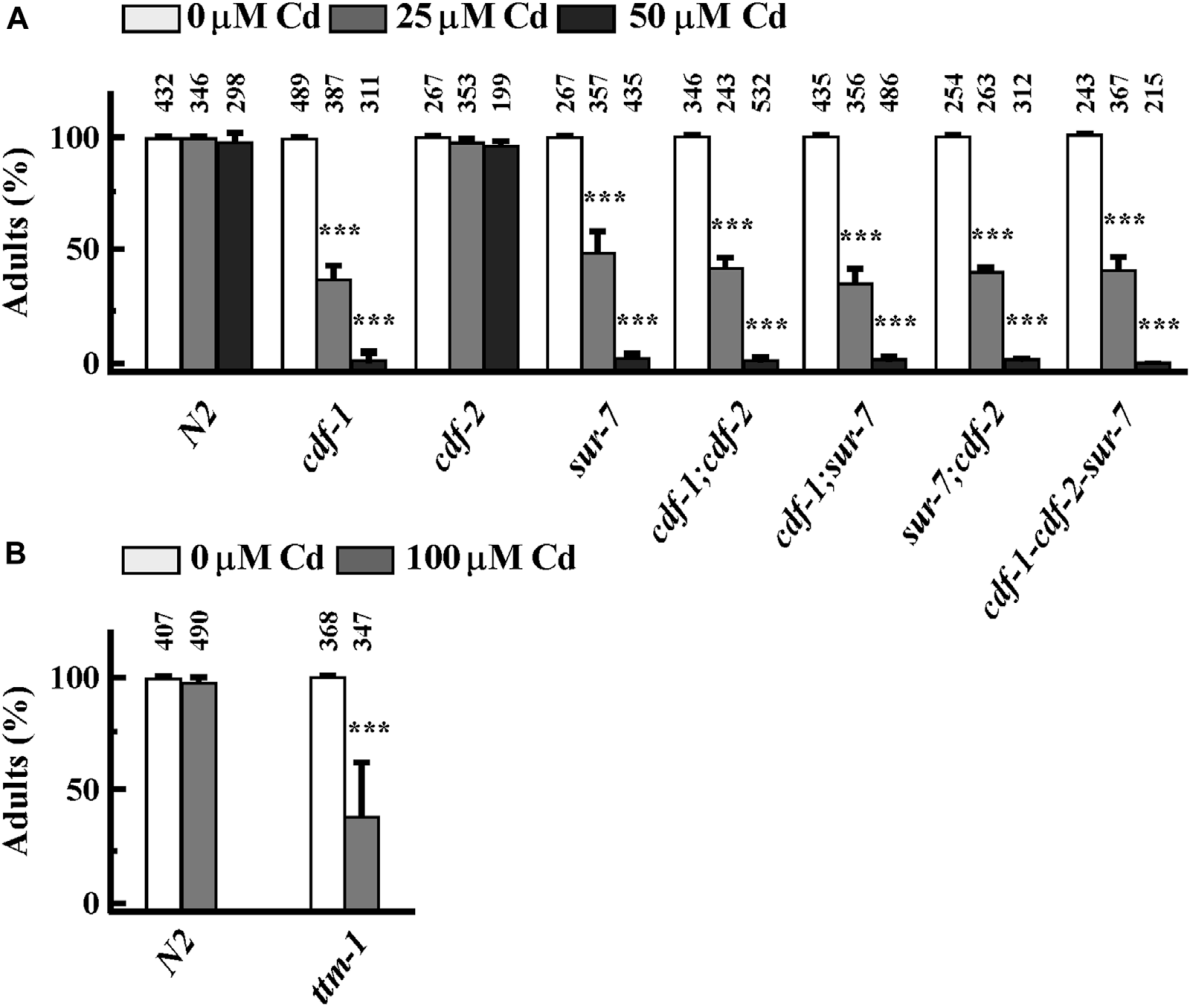
Zn transporters are involved in basal Cd resistance in *Caenorhabditis elegans*. **(A, B)** Percent of worms of indicated strains that reached the adult stage in the presence of an indicated concentration of CdCl_2_. The total number of worms tested is indicated above each bar. Error bars indicate ±S.E.M. The asterisks represent statistically significant differences between mean values of positive control, at 0 µM CdCl_2_ and Cd-treated conditions (*p < 0.05, **p < 0.01).

Cd enters the cells through various mechanisms. Cd uptake is mediated by Ca^2+^ channels in cultured mammalian cells (Hinkle and Osborne, 1994; Bressler et al., 2004a; Bergeron and Jumarie, 2006). The proton-coupled divalent metal ion transporter (DMT1) SLC11A2 shows a preference for Fe^2+^ but it also transports Pb^2+^ and Cd^2+^ (Bressler et al., 2004a). Similarly, Zn transporters also transport Cd in different human cell lines (Liu et al., 2008; Thevenod, 2010). The decreased accumulation of essential elements by Cd could be explained by the competition of Cd with other essential elements, sharing the same transporters/channels. During Cd-exposure, Zn and Cu transporters are considered the first choice; competition for uptake of Cd vs. Zn/Cu/Mn/Fe/Pb/Hg using *Xenopus* oocytes showed that Zn is the most efficient competitor of Cd, even with lower Zn/Cd ratio (Liu et al., 2008). In plants, the interaction between Zn and Cd is either antagonistic or synergistic depending on the plant tissue (Tkalec et al., 2014). Furthermore, a lower concentration of Zn decreased Cd-induced oxidative stress while a high concentration of Zn caused the accumulation of oxidative stress in plants (Tkalec et al., 2014). However, we favor the idea that Zn also plays a defensive molecular function in response to Cd toxicity at molecular level, in addition to competition with Cd for uptake, because we observed that Cd was still highly accumulated in the worm body when co-supplemented with Zn, where most of the tested worms were able to reach the adult stage (Figures 2A, I). Zinc oxide nanoparticles have also been shown to play Cd-remediation potential in maize (Tanveer et al., 2023). We further expanded our understanding of Cd and Zn interaction in *C. elegans*, revealing that Zn transporters (CDFs) and Zips genes play significant roles in Cd resistance. These transporters were highly upregulated by Cd, as confirmed by qRT-PCR analysis, and mutants lacking these transporters were hypersensitive to Cd (Figures 3, 4). This further suggests that maintaining Zn homeostasis is necessary in response to Cd toxicity. It is also possible that over-accumulation of Cd possibly mimics Zn accumulation and systematically triggered the hyper Zn response resulting in overexpression of Zn transporters even if Zn concentration is decreasing.

Zinc homeostasis in *C. elegans* is regulated by independent mechanisms depending on their amount. When Zn exists in high concentration, nuclear receptor transcription factor (HIZR-1) translocates to the nucleus in the intestine, and binds to HZA (high zinc activation) element in the promoter region of Zn homeostasis genes such as *ttm-1* and *cdf-2* for their activation (Warnhoff et al., 2017). When Zn is present in low concentration in *C. elegans*, zip genes such as *zipt-2.1, zipt-2.3* and *zipt-7.1* harboring LZA (low zinc activation) element in their promoter are highly activated by Zn deficiency, and require mediator subunit MDT-15 and GATA transcription factor ELT-2 (Dietrich et al., 2017). Further studies are required to elucidate the detailed mechanism of the interaction between Cd and Zn. It will be very informative to investigate how Cd affects Zn homeostasis mechanisms. Conducting carefully curated experimental tests involving the overexpression of select transporters, which emerged as significant from our analysis, under distinct treatment conditions involving Cd, Zn, and Cd-Zn, will greatly enhance our understanding of their contributions to the underlying process.

In contrast to Zn, Cu fortification in media could not rescue Cd toxicity rather led to enhanced necrosis and delayed development (Figures 2B, H). The homeostasis of Zn or Cu is important for the growth and developmental of worms, but their physiological tolerance and toxicity mechanisms are different (Chun et al., 2017; Davis et al., 2009). Due to its highly reactive nature, Cu stimulates free radical formation, causing severe damage to the cellular system, which is different from Cd (Takagi et al., 2002; Valko et al., 2005). Therefore, we speculate that decreased accumulation of Cu observed in worms treated with Cd may be a protective strategy in response to Cd accumulation. This scenario is different at least in some plant species. For example, in a model plant *Arabidopsis thaliana*, Cd toxicity is alleviated by Cu treatment (Gayomba et al., 2013). Unlike our studies in *C. elegans*, growing plants in the presence of Cd increases the expression of genes encoding Cu uptake transporters, *COPT1*, *COPT2*, and *COPT6* and stimulates Cu uptake *to A. thaliana roots* (Gayomba et al., 2013).

**FIGURE 5:**
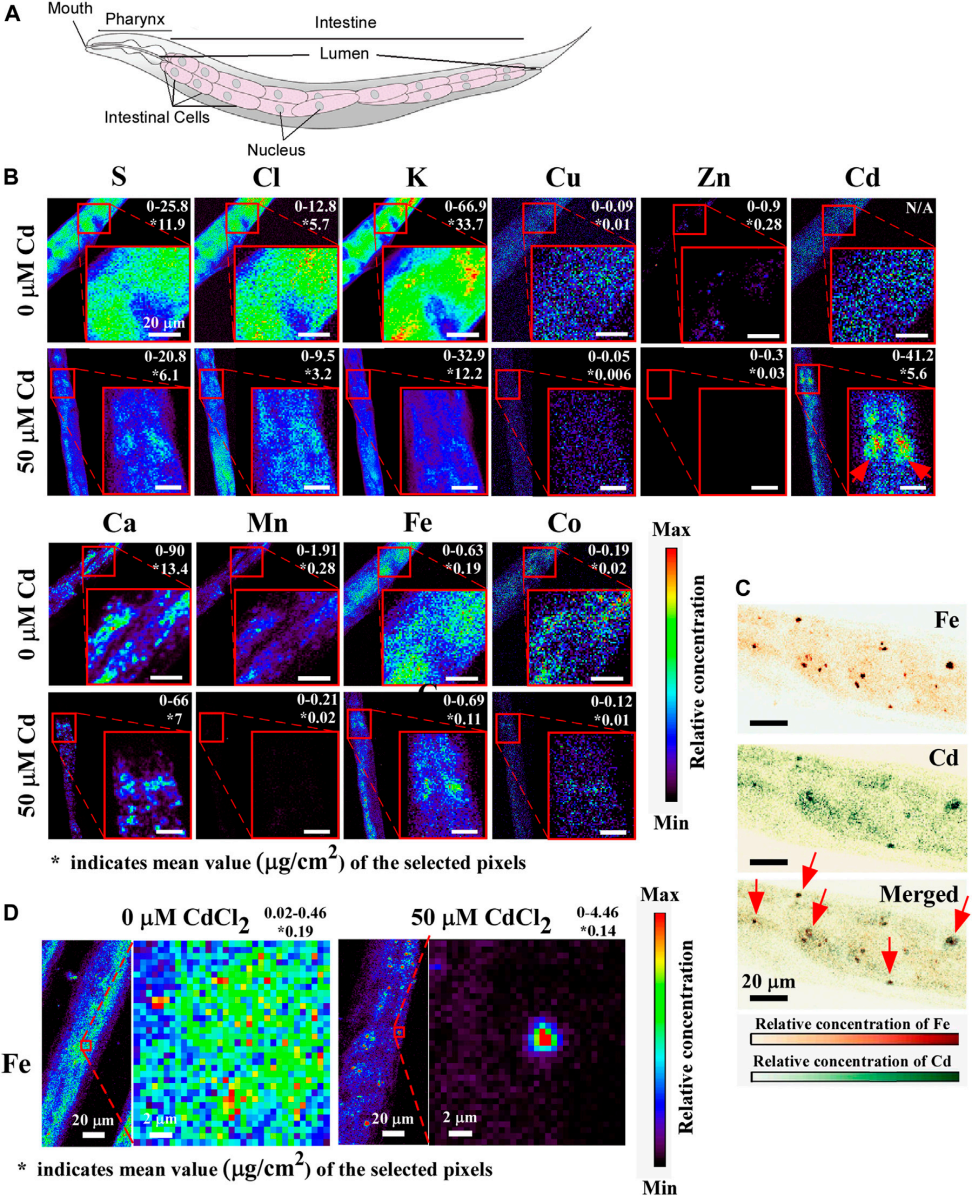
X-ray fluorescence microscopy (XRF) shows that Cd alters the concentration and distribution of essential elements. **(A)** A cartoon showing the arrangement of intestinal cells, adapted with permission from Herndon et al. (2018), licensed under CC BY 4.0. **(B)** Representative images of anterior intestinal cells showing the concentration and the distribution of sulfur (S), chlorine (Cl), potassium (K), copper (Cu), zinc (Zn), calcium (Ca), manganese (Mn), iron (Fe), Cobalt (Co) in the absence or presence of CdCl_2_. The value indicated on the top right corner represents the minimum to maximum concentration (µg/cm^2^) of the selected region. The red arrow indicated the accumulation of Cd in intestinal cells. The asterisk (*) represents the average concentration of the selected region of the indicated element. **(C)** Representative images of the magnified view of the distribution of Fe. **(D)** Representative images of Fe (upper) and Cd (middle) and merged (lower), shown with different color. Red arrows show that Fe and Cd are co-localized in intestinal cells to a vesicular structures. At least 5 worms were tested for 0 µM vs. 50 µM Cadmium condition and representative images are shown.

We discovered that the distribution of Fe was dramatically changed upon short-term exposure of Cd. Whereas Fe was evenly distributed in intestinal cells of worms grown without Cd, Fe gathered and hyper-accumulated and co-localized with Cd in punctate structures in the intestinal cells in Cd-grown worms (Figure 5). In this regard, our past studies have shown that Cd-treated worms, lacking ABCB6/HMT-1 transporter, accumulate refractile inclusions in intestinal cells and these inclusions associate with nuclei (Vatamaniuk et al., 2005a; Kim et al., 2018). These refractile inclusions, in Cd-grown *abcb-6/hmt-1* mutant, resemble Fe/Cd-containing puncta observed in this study (Figure 5). Interestingly, this abnormal Fe distribution is also observed in aged *C. elegans* (James et al., 2015; Jenkins et al., 2020). Twelve-day-old worms exhibited redistribution of Fe into vesicular inclusions in intestinal cells, in contrast to more disperse accumulation in four-day-old young worms (James et al., 2015). Studies in plants have shown that nucleolus is a hot spot for Fe-storing organelles in plants (Roschzttardtz et al., 2011). Whether Fe/Cd-accumulated puncta associate with the nucleus and whether the redistribution of Fe to these structures and its co-localization with Cd in Cd-grown *C. elegans* contribute to cytotoxicity are unknown and merit further investigations.

## Data Availability

The original contributions presented in the study are included in the article/Supplementary Material, further inquiries can be directed to the corresponding authors.
