# Supplementary material for: Cadmium alters whole animal ionome and promotes the re-distribution of iron in intestinal cells of Caenorhabditis elegans
Source: Front Physiol. 2023 Sep 26;14:1258540. doi: 10.3389/fphys.2023.1258540 (PMC10562743; doi:10.3389/fphys.2023.1258540)
Supplement: Supplementary file 1 [file DataSheet1.docx]

Supplementary Material

#
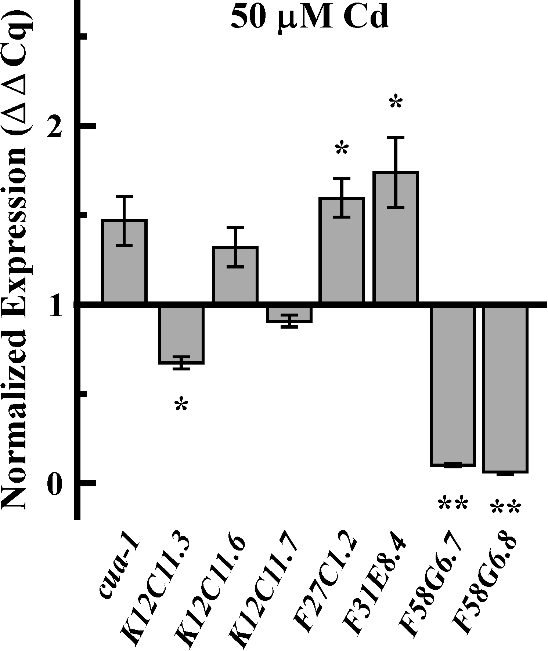
Supplementary Figure 1

#

**Figure S1.** RT-qPCR analysis revealed that Cd affects the transcriptional expression of Cu transporters and its homologs in *C. elegans* genome

# Supplementary Figure 2


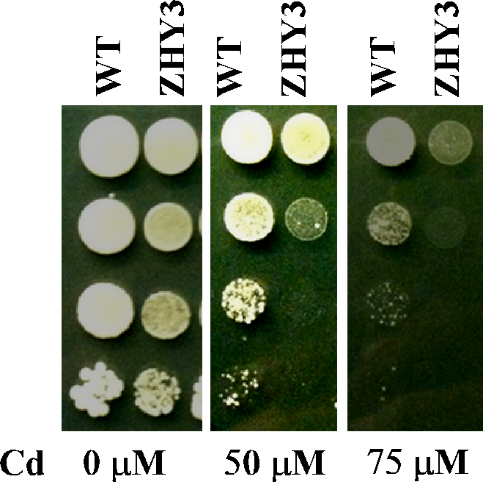


Figure S2. Zn transporters are involved in basal Cd resistance in S. cerevisiae yeast. Dilution series of wild type (WT) and *zrt-1;zrt-2* double mutant yeast cells (ZHY3) grown on the plates supplemented with indicated concentration of CdCl_2_.

# Supplementary Table S1

| **Supplemental Solution (1X Mineral Mix) for ICP-MS assays** | |
| --- | --- |
|  |  |
| **Reagent** | **Amount in 1 L** |
| MgCl_2_-6H_2_O | 6.15 g |
| sodium citrate | 4.35 g |
| potassium citrate-H_2_O | 7.35 g |
| CuCl_2_-2H_2_O | 0.105 g |
| MnCl_2_-4H_2_O | 0.3 g |
| ZnCl_2_ | 0.15 g |
| Fe(NH_4_)_2_(SO_4_)_2_-6H_2_O | 0.9 g |
| CaCl_2_-2H_2_O | 0.3 g |
| **From 1000 ppm stock** | **Final concentration in media** |
| **Al** | **500 ppb** |
| **Ni** | **400 ppb** |
| **As** | **1000 ppb** |
| **Sr** | **400 ppb** |
| **Mo** | **1000 ppb** |
| **Cd** | **50 ppb (0 Cd)**  **500 ppb (5 Cd)**  **5618 ppb (50 cd)** |
| **Rb** | **400 ppb** |
| **Co** | **50 ppb** |
| **Se [from K_2_SeO_4_]** | **5 ppm** |
|  |  |

Supplemental Solution (Mineral Mix) Adapted from Nass *et al*.,^1^

1. R. Nass and I. Hamza, The nematode C. elegans as an animal model to explore toxicology in vivo: solid and axenic growth culture conditions and compound exposure parameters, *Curr Protoc Toxicol*, 2007, Chapter 1, Unit1 9.

**
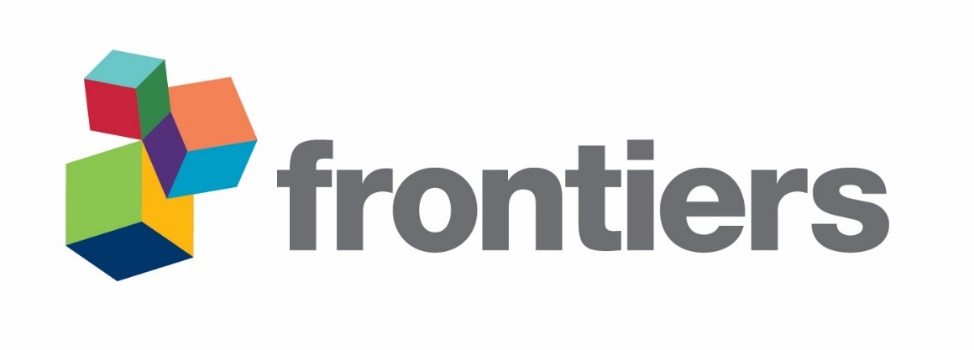
**
